# SARS-CoV-2 NSP13 helicase suppresses interferon signaling by perturbing JAK1 phosphorylation of STAT1

**DOI:** 10.1186/s13578-022-00770-1

**Published:** 2022-03-22

**Authors:** Sin-Yee Fung, Kam-Leung Siu, Huayue Lin, Ching-Ping Chan, Man Lung Yeung, Dong-Yan Jin

**Affiliations:** 1 School of Biomedical Sciences, The University of Hong Kong, 21 Sassoon Road, Pokfulam, Hong Kong, China; 2 Centre for Virology, Vaccinology and Therapeutics, Hong Kong Science and Technology Park, Hong Kong, China; 3 Department of Microbiology, The University of Hong Kong, 102 Pokfulam Road, Pokfulam, Hong Kong, China; 4 State Key Laboratory of Emerging Infectious Diseases, The University of Hong Kong, Pokfulam, Hong Kong, China; 5 Department of Clinical Microbiology and Infection Control, The University of Hong Kong-Shenzhen Hospital, Shenzhen, China

**Keywords:** SARS-CoV-2, COVID-19, NSP13, Helicase, JAK1, STAT1

## Abstract

**Background:**

SARS-CoV-2 is the causative agent of COVID-19. Overproduction and release of proinflammatory cytokines are the underlying cause of severe COVID-19. Treatment of this condition with JAK inhibitors is a double-edged sword, which might result in the suppression of proinflammatory cytokine storm and the concurrent enhancement of viral infection, since JAK signaling is essential for host antiviral response. Improving the current JAK inhibitor therapy requires a detailed molecular analysis on how SARS-CoV-2 modulates interferon (IFN)-induced activation of JAK-STAT signaling.

**Results:**

In this study, we focused on the molecular mechanism by which SARS-CoV-2 NSP13 helicase suppresses IFN signaling. Expression of SARS-CoV-2 NSP13 alleviated transcriptional activity driven by type I and type II IFN-responsive enhancer elements. It also prevented nuclear translocation of STAT1 and STAT2. The suppression of NSP13 on IFN signaling occurred at the step of STAT1 phosphorylation. Nucleic acid binding-defective mutant K345A K347A and NTPase-deficient mutant E375A of NSP13 were found to have largely lost the ability to suppress IFN-β-induced STAT1 phosphorylation and transcriptional activation, indicating the requirement of the helicase activity for NSP13-mediated inhibition of STAT1 phosphorylation. NSP13 did not interact with JAK1 nor prevent STAT1-JAK1 complex formation. Mechanistically, NSP13 interacted with STAT1 to prevent JAK1 kinase from phosphorylating STAT1.

**Conclusion:**

SARS-CoV-2 NSP13 helicase broadly suppresses IFN signaling by targeting JAK1 phosphorylation of STAT1.

## Background

The COVID-19 pandemic caused by SARS-CoV-2 has imposed a great burden on global health and healthcare system [1, 2]. Clinical manifestation of COVID-19 varies from asymptomatic or mild to severe disease [2, 3], including the life-threatening acute respiratory distress syndrome (ARDS) [3, 4]. Accumulating evidence suggests that cytokine release syndrome (CRS) is a root cause of ARDS in COVID-19, same as in the pathology of severe acute respiratory syndrome and the Middle East respiratory syndrome [5]. High levels of proinflammatory cytokines interleukin 2 (IL-2), IL-6 and IFN-γ were detected in sera of patients with severe COVID-19, suggesting that SARS-CoV-2 may usurp JAK-STAT signaling for pathogenesis [3, 5]. Complement system is also aberrantly activated in a JAK-dependent manner [6]. Multiple clinical trials are being conducted in COVID-19 patients to determine if the use of JAK inhibitors, such as ruxolitinib, baricitinib and tofacitinib, can alleviate CRS and inflammation [7–9]. JAK inhibitor therapy might introduce a trade-off between suppressing cytokine storm and sustaining antiviral response, as JAK signaling is primarily responsible for induction of IFN-stimulated genes (ISGs) that execute antiviral immunity [10]. To optimize JAK inhibition therapy, there is a need to better understand the molecular mechanism by which SARS-CoV-2 modulates IFN-induced JAK-STAT signaling.

To elicit antiviral response, autocrine and paracrine IFNs activate JAK-STAT signaling to induce ISG expression. Type I IFNs, such as IFN-α and IFN-β, trigger JAK1 and TYK2 phosphorylation upon binding to IFN-α/β receptors IFNAR1 and IFNAR2, leading to subsequent phosphorylation and heterodimerization of STAT1 and STAT2. The joining of IRF9 assembles the STAT1-STAT2-IRF9 complex, also known as transcription factor ISGF3, which translocates to the nucleus to activate IFN-stimulated response element (ISRE)-driven transcription of ISGs [10]. Type II IFN provokes STAT1 homodimerization through JAK1 and JAK2 phosphorylation, resulting in the stimulation of ISG transcription under the control of γ interferon activation sites (GAS) [10]. To achieve potent innate immune suppression, SARS-CoV-2 encodes various viral proteins to counteract IFN signaling. As a result, SARS-CoV-2 potently suppresses type I IFN response not only in cellular and animal models of infection but also in patients with severe COVID-19 [11–14]. SARS-CoV-2 infection prevents IFN-induced nuclear translocation of STAT1 and STAT2 [15]. Systematic screening with an expression library of SARS-CoV-2 proteins revealed that NSP1, NSP6, NSP13, NSP14, ORF3a, ORF6, ORF7a, ORF7b, matrix (M) and nucleocapsid (N) proteins antagonize type I IFN signaling [16–19]. NSP1 prevents ISG expression through shutdown of host translational machinery or downregulation of TYK2 and STAT2 expression [11, 20]. NSP6 impedes phosphorylation of STAT1 [17]. NSP14 induces lysosomal degradation of IFNAR1 [18]. ORF3a, M and N proteins inhibit IFN-β-induced activation of ISRE promoter [16, 17]. ORF6 suppresses nuclear translocation of STAT1 by hijacking Nup98 [15–17]. ORF7a blocks IFN-β-induced phosphorylation of STAT2, while N and ORF7b target phosphorylation of STAT1 and STAT2 [16, 21].

Helicase NSP13 of SARS-CoV-2 is essential for viral replication. Catalytically active as an NTPase and RNA helicase, NSP13 binds to RNA-dependent RNA polymerase to stimulate its backtracking [22]. It is also thought to facilitate proper folding and replication of viral RNA [23]. Inhibition of NSP13 with bismuth salts and other agents blocks viral replication [24–27]. In addition, NSP13 is capable of suppressing type I IFN production and signaling [17–19, 28, 29]. Its suppression of type I IFN production is mediated through the inhibition of phosphorylation and activity of TBK1-IKKε complex [17, 28, 29]. Up to date, several models have been proposed for NSP13-dependent suppression of type I IFN signaling. First, NSP13 blocks IFN-α-induced phosphorylation of STAT1 and STAT2 [17]. Second, NSP13 reduces the expression of IFNAR1 to exert a suppressive effect on type I IFN signaling [18]. Third, interactome profiling reveals an interaction between NSP13 and STAT1 [30]. Yet, the exact mechanism by which NSP13 antagonizes IFN signaling remains incompletely understood.

In this study, we investigated the molecular mechanism by which SARS-CoV-2 NSP13 antagonizes type I IFN signaling. NSP13 suppressed ISRE activation and STAT1 nuclear translocation induced by type I and type II IFNs. NSP13 targeted STAT1 phosphorylation but did not affect kinase activity of JAK1. NSP13 neither interacted with JAK1, nor impeded JAK1-STAT1 complex formation. Instead, NSP13 interacted with STAT1 to prevent it from phosphorylation by JAK1. Mutational analysis of NSP13 revealed the essentiality of helicase activity in NSP13-dependent suppression of type I IFN signaling.

## Results

### Suppression of IFN response by SARS-CoV-2 NSP13

**Fig. 1:**
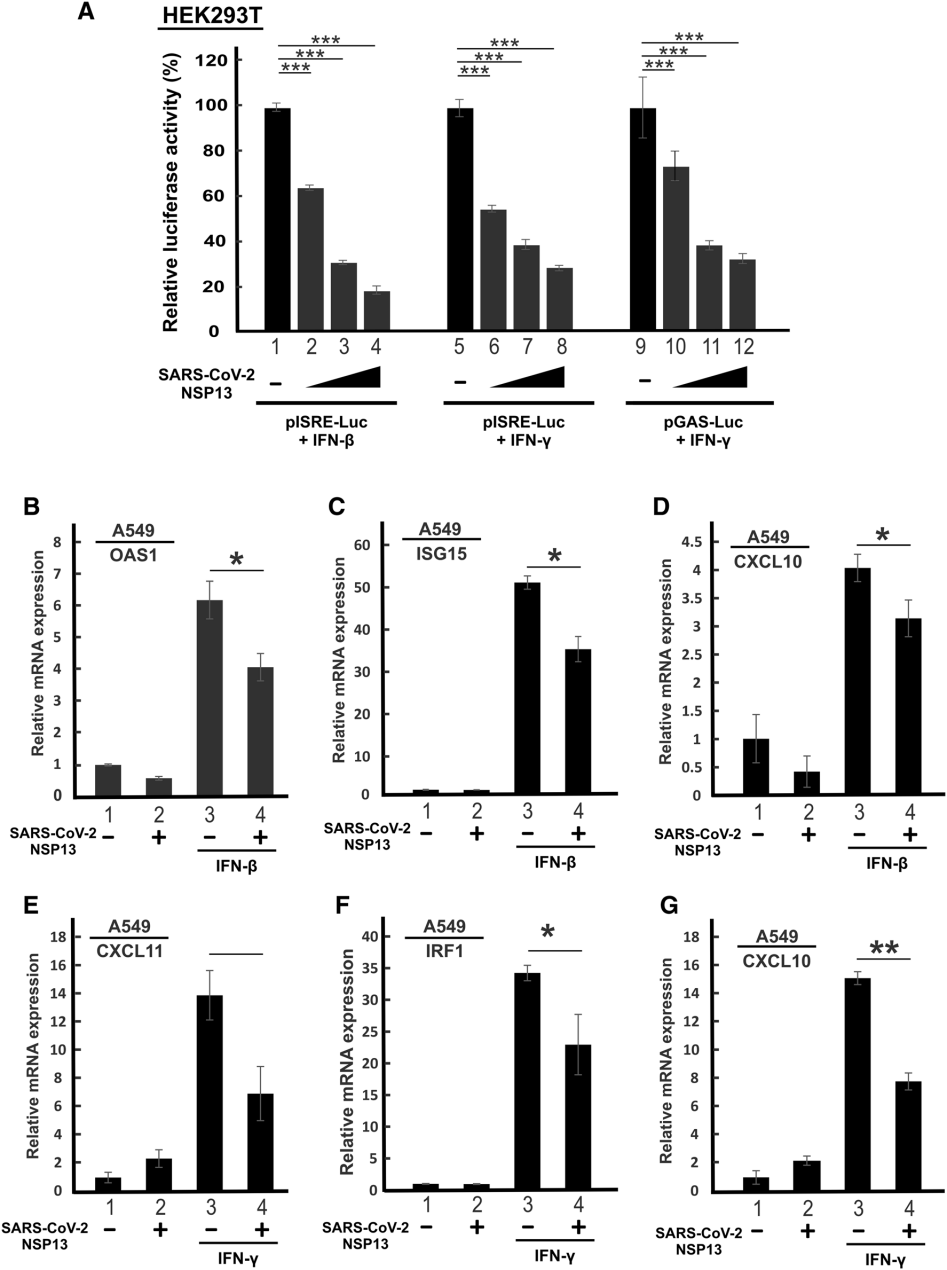
SARS-CoV-2 NSP13 suppresses type I and type II IFN signaling. **A** NSP13 suppresses IFN-β- and IFN-γ- induced ISRE and GAS promoter activity. HEK293T cells were transfected with pISRE-Luc or pGAS-Luc, SV40 Renilla luciferase and increasing doses of NSP13 plasmid (200, 400 and 600 ng). At 24 h post transfection, cells were stimulated by 1000 U/mL of IFN-β or 100 ng/mL of IFN-γ. Dual luciferase activity was measured 24 h post IFN treatment. **B**–**G** Suppression of IFN-β and IFN-γ signaling by NSP13. A549 cells were either mock transfected or transfected with NSP13 plasmid. At 26 h post transfection, cells were stimulated by 1000 U/mL IFN-β or 100 ng/mL IFN-γ for 6 h. ISG transcripts were analyzed by RT-qPCR. Results were representative of three independent experiments. The statistical significance of the differences between the indicated groups was evaluated by one-tailed Student t test for unpaired samples with equal variance. *P < 0.05. **P < 0.01. ***P < 0.001

Antagonism of type I IFN signaling by SARS-CoV-2 NSP13 has been demonstrated in several studies [16–18, 30]. Consistent with this, we observed dose-dependent suppression of IFN-β-induced activation of ISRE-driven luciferase expression by NSP13 in HEK293T cells (Fig. 1A, lanes 1–4). HEK293T cells were chosen for mechanistic study on SARS-CoV-2 due to their high transfection efficiency and susceptibility to SARS-CoV-2 infection particularly after introduction of ACE2 and TMPRSS2 [31]. Diminution of type I and type II IFN production and response in patients with severe COVID-19 [32, 33] raised the possibility that SARS-CoV-2 might also antagonize type II IFN signaling. We therefore investigated further whether NSP13 specifically counteracts type II IFN signaling. When we stimulated ISRE-driven luciferase expression with IFN-γ in the presence of progressively increasing doses of NSP13 expression plasmid, a dose-dependent attenuation of IFN-γ-induced activation of ISRE-dependent transcription was seen (Fig. 1A, lanes 5–8). When we repeated the assay with pGAS-Luc reporter construct driven by the IFN-γ-responsive GAS enhancer element, similar suppressive effect by NSP13 was observed (Fig. 1A, lanes 9–12).

Bearing in mind that SARS-CoV-2 primarily targets cells in the respiratory tract [4], we further interrogated whether expression of NSP13 might also suppress IFN-β- and IFN-γ-induced ISG expression in A549 cells, a lung carcinoma cell line susceptible to SARS-CoV-2 infection [17]. Whereas the transcription of antiviral genes ISG15 and OAS1 is primarily regulated by type I IFNs, expression of IRF1 and CXCL11 is governed by type II IFN. In addition, CXCL10 can be induced by both type I and type II IFNs. As expected, treatment with IFN-β induced the expression of ISG15 and OAS1 transcripts (Fig. 1B, C, bar 3 versus 1), while treatment with IFN-γ boosted CXCL11 and IRF1 mRNA expression (Fig. 1E, F, bar 3 versus 1). The expression of CXCL10 mRNA was stimulated by both IFN-β and IFN-γ (Fig. 1D and G, bar 3 versus 1). In line with our above observation on ISRE- and GAS-driven reporter assay, ectopic expression of NSP13 antagonized IFN-β- and IFN-γ-induced ISG expression in A549 cells (Fig. 1B–G, bar 3 versus 4). Collectively, our results are in general agreement with the recent finding that SARS-CoV-2 NSP13 suppresses both type I and type II IFN signaling [18, 30].

### Suppression of IFN-β- and IFN-γ-induced nuclear translocation of STAT1 and STAT2 by NSP13

**Fig. 2:**
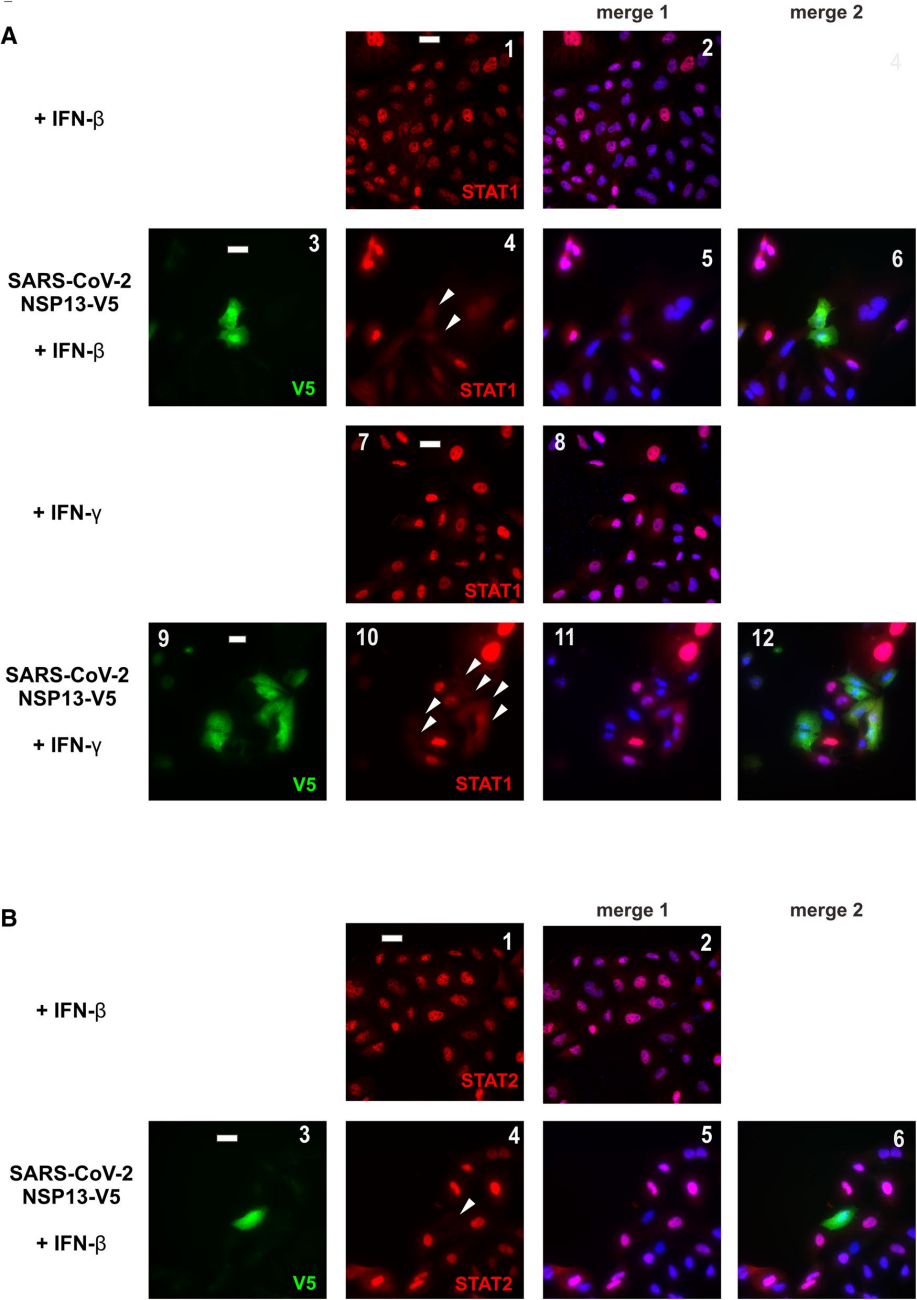
SARS-CoV-2 NSP13 prevents nuclear translocation of STAT1 and STAT2. **A** NSP13 blocks IFN-β- and IFN-γ-induced nuclear translocation of STAT1. A549 cells were transfected with an expression plasmid for V5-tagged NSP13. At 24 h post transfection, transfected cells were stimulated by 1000 U/mL IFN-β or 100 ng/mL IFN-γ for 30 min, and then stained for total STAT1 (red) and V5 (green). 4′, 6-diamidino-2-phenylindole (DAPI, blue) was used to visualize nuclear morphology. Transfected cells were highlighted by arrows. STAT1 and DAPI signals were merged in panels 2, 5, 8 and 11, while STAT1, NSP13 and DAPI signals were merged in panels 6 and 12. **B** NSP13 counteracts IFN-β-induced nuclear translocation of STAT2. Endogenous STAT2 was in red. STAT2 and DAPI signals were merged in panels 2 and 5, while STAT2, NSP13 and DAPI signals were merged in panel 6. Bar, 20 µm. Results were representative of three independent experiments

To determine how SARS-CoV-2 NSP13 suppresses type I and type II IFN response, we next addressed whether NSP13 might be influential on the subcellular localization of transcription factors STAT1 and STAT2 upon IFN treatment. NSP13 was ectopically expressed in A549 cells for 24 h before cells were treated with IFN-β or IFN-γ. Nuclear localization of endogenous STAT1 and STAT2 was then observed. Both STAT1 and STAT2 translocated to the nucleus when cells were stimulated with IFN-β (Fig. 2A, B, panels 1–2), but only STAT1 did so in cells treated with IFN-γ (Fig. 2A, panels 6–7). When ectopic expression of NSP13 was enforced, STAT1 and STAT2 were diffusely distributed in the cytoplasm (Fig. 2A, B, arrowed cells in panels 4 and 8). These results are compatible with the suppression of IFN-induced nuclear translocation of STAT1 and STAT2 by NSP13.

**Fig. 3:**
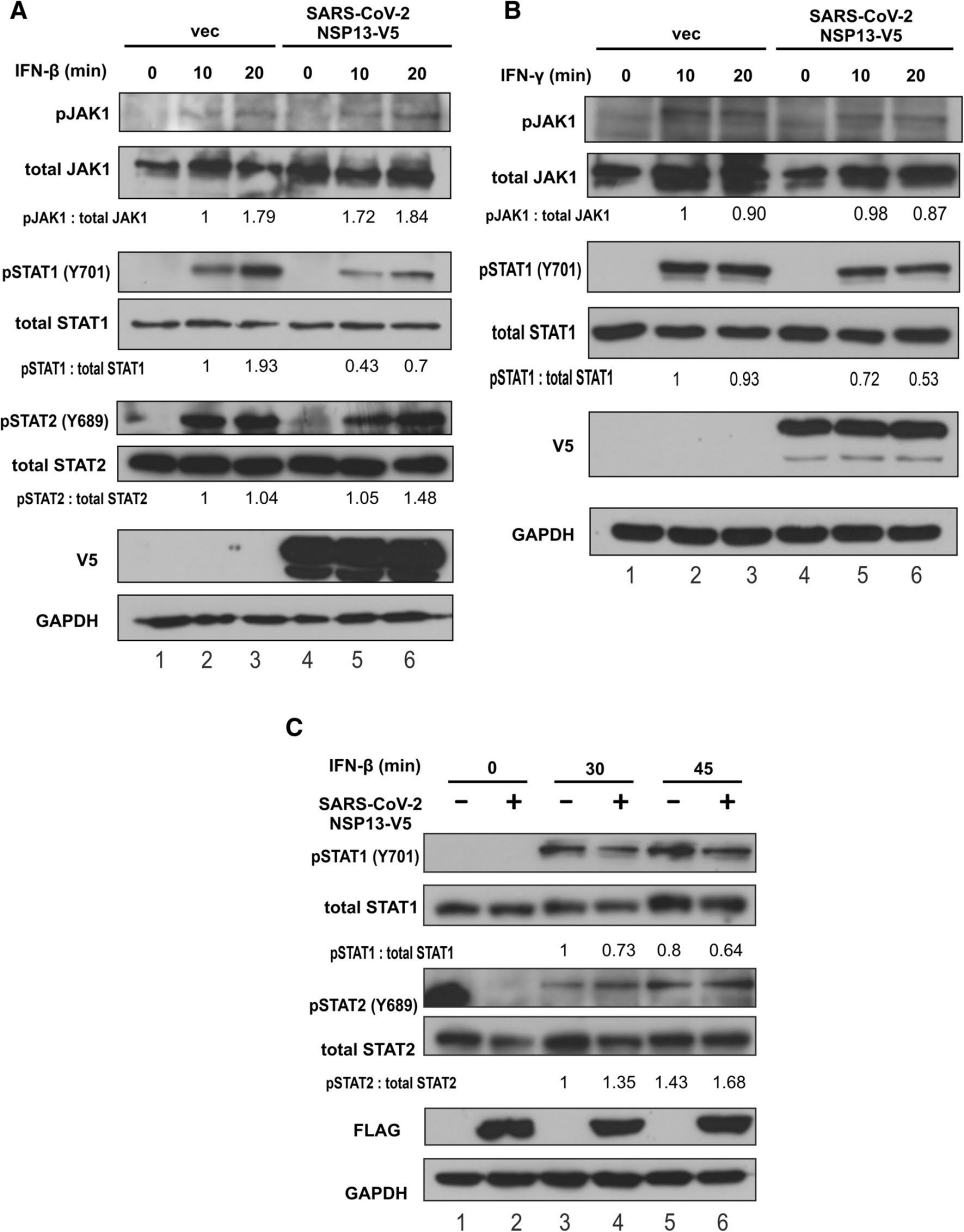
SARS-CoV-2 NSP13 suppresses type I and II IFN signaling by preventing STAT1 phosphorylation. HEK293T cells were transfected with an expression plasmid for V5-tagged NSP13. **A** At 48 h post transfection, cells were stimulated by 1000 U/mL of IFN-β. **B** Cells were treated with 100 ng/mL of IFN-γ. At 10- and 20-min post IFN treatment, cell lysates were collected and analyzed by Western blotting with the indicated antibodies. **C** Experiment in A was repeated with FLAG-tagged NSP13. At 30 and 45 min post IFN-β treatment, cell lysates were collected and analyzed by Western blotting. Relative ratios of pSTAT1 versus total STAT1, pSTAT2 versus total STAT2 and pJAK1 versus total JAK1 were determined by densitometry and are indicated below the blots. pSTAT1: phospho-STAT1. pSTAT2: phospho-STAT2. pJAK1: phospho-JAK1. Results were representative of three independent experiments

To derive additional mechanistic insight into NSP13 suppression of IFN signaling, we sought to identify the target of NSP13 in the JAK-STAT pathway. We hypothesized that NSP13 might target JAK1 kinase or STAT1 transcription factor, either of which is shared by type I and type II IFN signaling pathways. In this regard, Zika virus helicase NS2B3 is known to suppress type I IFN signaling through proteasomal degradation of JAK1 [34]. We first monitored the phosphorylation status and protein expression levels of JAK1 and STATs. Since nuclear translocation of STATs was seen after treatment with IFN-β for 30 min, phosphorylation of JAK1 and STATs was observed within 10 and 20 min after IFN-β treatment. JAK1 and STAT1 phosphorylation was evident upon IFN-β and IFN-γ stimulation, whereas STAT2 phosphorylation was seen in IFN-β stimulated cells (Fig. 3A, B, lanes 2 and 3 compared to lane 1). Expression of NSP13 had no influence on IFN-β-induced JAK1 phosphorylation, nor did it affect protein expression of JAK kinase (Fig. 3A, lanes 5 and 6 compared to lane 4). In IFN-γ-treated cells, JAK1 phosphorylation was unchanged or marginally reduced upon NSP13 expression (Fig. 3B, lanes 4–6). Hence, NSP13 was unlikely influential on JAK1 expression or phosphorylation. Instead, NSP13 impaired IFN-β- and IFN-γ-induced accumulation of phospho-STAT1, but it did not reduce the levels of phospho-STAT2 (Fig. 3A, B, lanes 4–6). The reduction of phospho-STAT1 in the presence of NSP13 was also observed in later time points (Fig. 3C, lanes 4–6). Thus, NSP13 suppresses type I and type II IFN signaling by inhibiting STAT1 phosphorylation.

### Prevention of JAK1 phosphorylation of STAT1 by NSP13

**Fig. 4:**
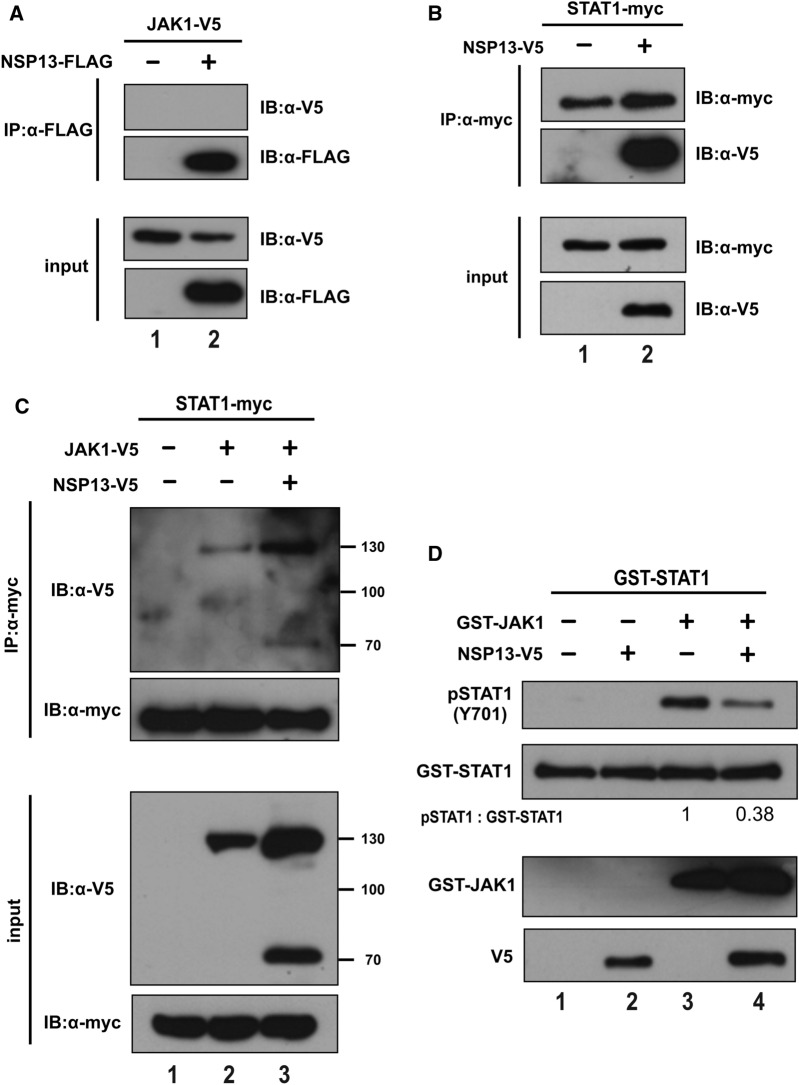
SARS-CoV-2 NSP13 interacts with STAT1 to prevent it from being phosphorylated by JAK1. **A**, **B** NSP13 interacts with STAT1 but not JAK1. HEK293T cells were transfected with vectors expressing the indicated proteins. Immunoprecipitation (IP) was performed 48 h post transfection. NSP13 was immunoprecipitated with anti-FLAG or anti-V5. Precipitates were probed with anti-V5, anti-FLAG or anti-myc. **C** NSP13 does not impede complex formation between JAK1 and STAT1. HEK293T cells were transfected with plasmids expressing the indicated proteins. Immunoprecipitation were performed at 48 h post transfection. STAT1 was immunoprecipitated with anti-myc. Precipitates were probed with anti-V5 and anti-myc. **D** NSP13 blocks STAT1 phosphorylation by JAK1. HEK293T cells was transfected with expression plasmid for V5-tagged NSP13. At 48 h post-transfection, NSP13 protein was immunoprecipitated with anti-V5. The precipitates were incubated with recombinant STAT1 and JAK1 in the presence of 10 mM ATP for 30 min at 30 °C. Phospho-STAT1 (pSTAT1) was probed with anti-pSTAT1 antibodies. Results were representative of three independent experiments

NSP13 prevented IFN-β- and IFN-γ-induced phosphorylation of STAT1 without affecting its steady-state expression (Fig. 3). To dissect how NSP13 might inhibit STAT1 phosphorylation, we interrogated whether NSP13 interacted with JAK1 or STAT1. We overexpressed NSP13, JAK1 and STAT1 in HEK293T cells to perform co-immunoprecipitation. The absence of JAK1 in the NSP13 precipitate and the presence of NSP13 in the STAT1 precipitate indicated interaction of NSP13 with STAT1 but not JAK1 (Fig. 4A, B, lane 2 compared to lane 1).

The association of NSP13 with STAT1 raised two possible explanations for NSP13 inhibition of STAT1 phosphorylation. First, NSP13 might prevent phosphorylation of STAT1 by competing with JAK1 for binding with STAT1 and thereby impeding JAK1-STAT1 complex formation. Second, NSP13 might prevent STAT1 from phosphorylation by JAK1. To distinguish between the two possibilities, we first determined whether JAK1-STAT1 complex formation was compromised upon expression of NSP13. Surprisingly, JAK1-STAT1 interaction was pronounced when STAT1, JAK1 and NSP13 were overexpressed in HEK293T cells (Fig. 4C, lane 3 compared to 2). Thus, NSP13 is not competing with JAK1 for STAT1 binding.

We further investigated whether NSP13 might suppress JAK1 kinase activity on STAT1. We expressed and immunoprecipitated NSP13 from HEK293T cells (Fig. 4D). The precipitate was incubated with recombinant GST-JAK1 and GST-STAT1 proteins. Kinase activity of JAK1 was analyzed by levels of phospho-STAT1 by Western blotting. Notably, NSP13-containing precipitate inhibited JAK1 phosphorylation of STAT1 (Fig. 4D, lane 4 compared to lane 3). Thus, NSP13 was capable of suppressing JAK1 kinase activity on STAT1, leading plausibly to inhibition of STAT1 activation and subsequent IFN signaling.

### Requirement of helicase activity for NSP13 suppression of IFN signaling

**Fig. 5:**
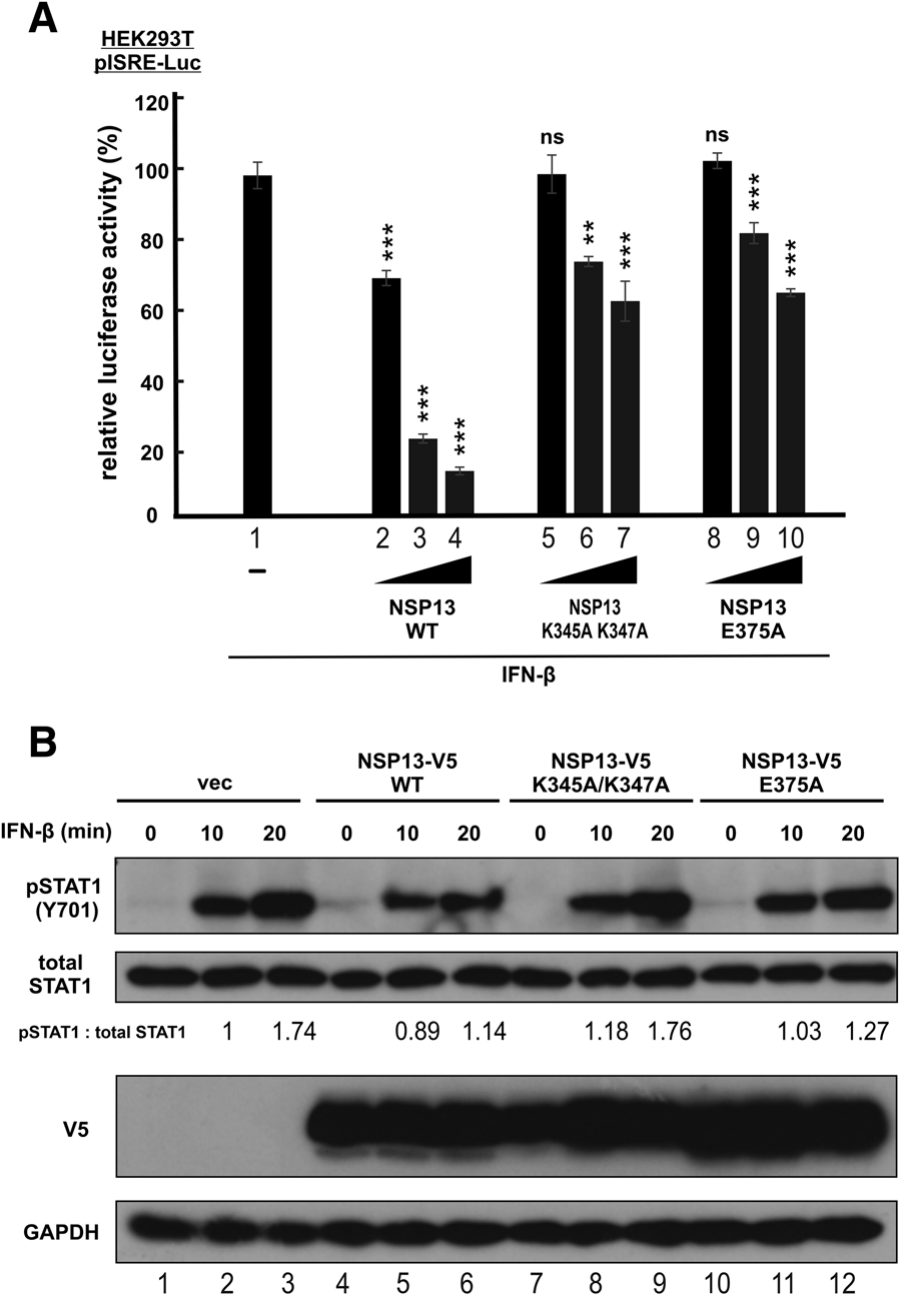
Helicase activity of SARS-CoV-2 NSP13 is required for suppression of type I IFN signaling. **A** Experiments in Fig. 1 were repeated with nucleic acid binding-defective mutant K345A K347A and NTP binding-defective mutant E375A of NSP13. The statistical significance for the differences between the indicated group and the empty vector control group (group 1) was determined by a one-tailed Student t test for unpaired samples with equal variance. ns: P ≥ 0.05 (not significant). **P < 0.01. ***P < 0.001. **B** Experiments in Fig. 3A were repeated with the indicated NSP13 mutants. Relative ratios of pSTAT1 to total STAT1, were determined by densitometry and are indicated below the blots. pSTAT1: phospho-STAT1. Results were representative of three independent experiments

RNA helicase activity of NSP13 is essential for SARS-CoV-2 replication [22, 23]. With this in mind, we asked whether helicase activity of NSP13 might also be required for its suppressive activity on IFN signaling. To this end, we created two mutants of NSP13, nucleic acid binding-defective mutant K345A K347A and NTP binding-defective mutant E375A [35]. IFN-β-induced activation of ISRE-driven transcriptional activity was partially restored when these mutants were expressed (Fig. 5A, bars 5–7 and 8–10 compared to bars 2–4). Whereas K345A K347A mutant of NSP13 failed to suppress IFN-β-induced STAT1 phosphorylation, E375A mutant exhibited a partial suppressive effect on STAT1 phosphorylation after prolonged treatment with IFN-β for 20 min (Fig. 5B, lane 12 compared to lanes 11 and 3). Considered together with the previous finding that the helicase activity is incompletely inhibited in these mutants [35], our results suggested the requirement of helicase activity of NSP13 for its suppression of IFN signaling.

## Discussion

SARS-CoV-2 NSP13 is a viral protein that suppresses IFN production and signaling at multiple steps [17, 18, 28–30]. NSP13 is thought to antagonize IFN signaling by preventing IFN-β-induced phosphorylation of STAT1 and STAT2 [17], reducing endogenous IFNAR expression [18], and interacting with STAT1 [30]. Our study revealed additional mechanistic details for NSP13-dependent suppression of IFN signaling. Particularly, NSP13 interacts with STAT1. It does not affect STAT1-JAK1 interaction, but it inhibits JAK1 kinase activity on STAT1. Although the suppression of type I IFN signaling by SARS-CoV-2 NSP13 has been well documented [16–18, 28–30], our refined model provides an explanation for the suppressive effect of NSP13 on both type I and type II IFN signaling.

To evade host immune surveillance, many viruses have evolved to subvert both type I and II IFN signaling by targeting JAKs and STATs [36]. For example, Sendai virus C protein mitigates IFN-α and IFN-γ signaling by suppressing STAT1 phosphorylation or degrading STAT1 [37, 38]. Measles virus and Hendra virus V protein suppresses type I and type II IFN signaling by preventing STAT1 and STAT2 nuclear translocation [39, 40]. Our demonstration of NSP13 perturbation of IFN-induced STAT1 phosphorylation and nuclear translocation not only suggests a new model for its mechanism of action, but also raises interesting questions that require further investigations. NSP13 interacts with STAT1 and prevents it from phosphorylation by JAK1. Addition of NSP13 to the mixture of recombinant JAK1 and STAT1 resulted in the inhibition of JAK kinase activity on STAT1. Since NSP13 does not compete with JAK1 for binding with STAT1 (Fig. 4C) or affect JAK1 autophosphorylation (Fig. 3), its inhibition of JAK1 phosphorylation of STAT1 might be specific and requires binding with STAT1. Based on this, it is tempting to suggest a working model in which NSP13 specifically affects JAK1 phosphorylation of STAT1 by binding with STAT1 to render the JAK1 phosphorylation site inaccessible, plausibly through creating steric hindrance or inducing conformational change. In this regard, it will be of great interest to determine whether NSP13, JAK1 and STAT1 form a triple complex, whether NSP13 binding to STAT1 is required for its suppressive activity on JAK1, and how NSP13 binding induces conformational change of STAT1. Several lines of new experiments including co-immunoprecipitation, mutational analysis and structural analysis might be required. Detailed domain mapping should also be performed to define the key residues and regions in STAT1 and NSP13 that mediate their interaction. Furthermore, experiments shown in Fig. 4D should be repeated with more controls to determine whether and how NSP13 influences JAK1 phosphorylation of substrates other than STAT1 and JAK1.

In this report, we only explored how NSP13 circumvents canonical type I and type II IFN signaling. Due to the complexity of JAK-STAT signaling, many unanswered questions about the impact of NSP13 on non-canonical IFN signaling await further investigations. First, IKKε can phosphorylate STAT1 [41]. Several studies have suggested a suppressive effect of NSP13 on IKKε [17, 28, 29]. It will be intriguing to clarify whether NSP13 might also affect IFN signaling by preventing IKKε-dependent STAT1 activation. Second, noncanonical activation of JAK-STAT signaling should be explored. Both type I and type II IFNs induce CRKL phosphorylation and subsequent CRKL-STAT5 signaling for ISG transcription [10]. In this regard, it is interesting to test if NSP13 also prevents STAT5 signaling. Third, a board range of cytokines induced in patients with severe COVID-19, including IL-2, IL-7 and IL-6 family cytokines, employ JAK1 for signal transduction [3, 42, 43]. It is crucial to determine if NSP13 causes aberrant induction of JAK-STAT signaling upon stimulation with other cytokines.

NSP13 has previously been shown to suppress IFN-α-induced phosphorylation of STAT1 and STAT2 [17]. It has also been suggested to inhibit the expression of endogenous IFNAR1 but have no influence on IFN-β-induced phosphorylation of STAT1 or STAT2 [18]. Since the suppressive effect of NSP13 on STAT2 phosphorylation or IFNAR1 expression observed in the previous two studies [17, 18] is relatively mild, it might not account for the broad inhibition of IFN signalling by NSP13 in full. Particularly, suppression of neither STAT2 [17] nor IFNAR1 [18] can explain the reported inhibitory effect of NSP13 on type II or III IFN signaling [30]. In this regard, our model based on selective inhibitory effect of NSP13 on the common step of JAK1 phosphorylation of STAT1 provides a new explanation of the IFN antagonism of NSP13. Further investigations are required to resolve the discrepancies and to clarify whether the suppression of JAK phosphorylation of STAT1 by NSP13 would play a major role in the IFN-antagonizing property of SARS-CoV-2.

The helicase activity of NSP13 is required for optimal suppression of IFN signaling (Fig. 5). To what extend its IFN antagonism might contribute to the inviable phenotype of NSP13-deficient SARS-CoV-2 (our unpublished data) merits further elucidation. If IFN antagonism is critical, the NSP13-deficient virus might be rescued in IFN-deficient cells such as Vero. Two molecules of NSP13 bound to RNA are found in the replication and transcription complex of SASR-CoV-2 [44]. It will be of interest to clarify whether and how its interaction with STAT1 might affect the function of NSP13 in viral replication.

Conserved NSP13 protein sequence among CoVs and absolute requirement of NSP13 for viral replication suggest that NSP13 is an important target for design of antiviral drugs [44–47]. We demonstrated that helicase activity is required for NSP13 antagonism of type I IFN signaling. Since coronaviral helicases are highly conserved, it will be of interest to see if STAT1 suppression by SARS-CoV-2 NSP13 represents a common strategy for IFN signaling antagonism across CoVs. Further mechanistic analysis of NSP13 suppression of STAT1 through structural determination of NSP13-STAT1 complex may pave the avenue for drug development including functional screening of compound libraries in search of NSP13 inhibitors [25, 26, 48, 49].

## Conclusions

SARS-CoV-2 NSP13 a pan antagonist of IFN signaling that prevents IFN-β- and IFN-γ-induced activation of ISRE-driven transcription as well as STAT1 nuclear translocation and phosphorylation. NSP13 interacts with STAT1 but not JAK1. Instead of impeding JAK1-STAT1 complex formation, NSP13 suppresses JAK1 phosphorylation of STAT1.

## Methods

### Plasmids

Mammalian expression constructs of STAT1 and STAT2 were described elsewhere [50]. Mammalian expression construct of JAK1 was constructed by standard molecular cloning technique. Luciferase reporter constructs pISRE-Luc and pGAS-Luc were from Promega. SARS-CoV-2 NSP13 cDNA was amplified and cloned from cells infected with SARS-CoV-2 [51]. Point mutants of SARS-CoV-2 NSP13 were constructed by Q5® Site-Directed Mutagenesis Kit (New England Biolabs, MA, USA).

### Cell lines and transfection

HEK293T and A549 cells were grown in Dulbecco's Modified Eagle Medium supplemented with 10% fetal bovine serum and 100 U/ml of penicillin/streptomycin (Thermo Fisher Scientific, MA, USA) in humidified chamber at 37 °C, supplemented with 5% CO_2_. HEK293T cells were transfected with Genejuice reagents (MilliporeSigma, MA, USA). A549 cells were transfected by use of Lipofectamine 3000 (Thermo Fisher Scientific). IFN-β1a and IFN-γ were purchased from PBL Assay Science (NJ, USA).

### Luciferase assay and protein analysis

Dual luciferase assay, confocal immunofluorescence microscopy, immunoprecipitation and Western blotting were performed as previously described [50–52]. Relative luciferase activity in arbitrary units was calculated by normalizing firefly luciferase activity with Renilla luciferase activity.

Mouse anti-c-myc (clone 9E10) was purchased from MilliporeSigma. Mouse anti-V5 and anti-GST were from Thermo Fisher Scientific. Rabbit anti-STAT1, rabbit anti-STAT-2, mouse anti-JAK1 (A-9), and mouse anti-GAPDH were purchased from Santa Cruz Biotechnology (TX, USA). Rabbit polyclonal antibodies against phospho-STAT1, phospho-JAK1 were bought from Cell Signaling Technology (MA, USA). Anti-phospho-STAT2 was purchased from R&D Systems (MN, USA).

### RT-qPCR

RT-qPCR was performed as previously described [50]. In brief, total RNA was extracted with RNeasy Mini Kit (Qiagen, Hilden, Germany). Genomic DNA was removed and cDNA was synthesized with PrimeScript RT Reagent Kit with gDNA Eraser (TaKaRa Bio, Kusatsu, Japan). Real-time PCR was performed with TB green Premix Ex Taq reagents (TaKaRa) and CFX96 Touch Real-Time PCR Detection System (Bio-Rad, CA, USA). The normalized value for each sample was derived from the relative quantity of target mRNA divided by the relative quantity of hypoxanthine phosphoribosyl transferase 1 (HPRT1) mRNA. The primers for ISG15, OAS1, CXCL10, IRF1 have been described previously [50]. Other primers were 5′- GACGCTGTCTTTGCATAGGC-3′ and 5′-GGATTTAGGCATCGTTGTCCTTT-3′ for CXCL11; 5′- TGACACTGGCAAAACAATGCA-3′ and 5′- GTCCTTTTCACCAGCAAGCT-3′ for HPRT1.

### In vitro kinase assay

Kinase activity was assayed in vitro as described [53]. Briefly, NSP13 were immunoprecipitated from transfected HEK293T cells. The precipitates were resuspended in kinase buffer (5 mM Tris–HCl (pH 7.5), 5 mM β-glycerophosphate, 2 mM dithiothreitol, 0.1 mM Na_3_VO_4_ and 10 mM MgCl_2_) and incubated with 10 mM ATP, recombinant STAT1 (Sino Biological) and recombinant JAK1 (MilliporeSigma) at 30 °C for 30 min. Reaction was stopped by addition of 5 × protein sample buffer and samples were analyzed by SDS-PAGE followed by Western blotting.

## Data Availability

All datasets used and/or analyzed during the current study are available from the corresponding author upon reasonable request.

## Abbreviations

ARDS: Acute respiratory distress syndrome
ATP: Adenosine triphosphate
cDNA: Complementary DNA
CoV: Coronavirus
COVID-19: Coronavirus Disease 2019
CRS: Cytokine release syndrome
CXCL: C-X-C motif chemokine ligand
GAS: γ Interferon activation site
HPRT1: Hypoxanthine phosphoribosyl transferase 1
IFN: Interferon
IFNAR: Interferon-α/β receptor
IKK: IκB kinase
ISG: Interferon-stimulated gene
ISRE: Interferon-stimulated response element
IL: Interleukin
IRF: Interferon regulatory factor
JAK: Janus kinase
NTP: Nucleoside triphosphate
OAS1: 2′-5′-Oligoadenylate synthetase 1
STAT: Signal transducer and activator of transcription
TBK1: TANK-binding kinase 1
